# Prognostic significance of stem cell marker CD133 determined by promoter methylation but not by immunohistochemical expression in malignant gliomas

**DOI:** 10.1007/s11060-015-2039-z

**Published:** 2016-01-12

**Authors:** Xing Wu, Fenlang Wu, Dongwen Xu, Tao Zhang

**Affiliations:** Department of Neurosurgery, Huashan Hospital, Fudan University, Shanghai, China; Department of Neurosurgery, Yangjiang Hospital, Guangdong Medical College, Guangzhou, China; Department of Laboratory Medicine, Huashan Hospital, Fudan University, 12 Central Urumqi Road, Shanghai, 200040 China

**Keywords:** CD133, Gliomas, Promoter methylation, Patient outcome, Brain tumor stem cells

## Abstract

CD133 has played a pivotal role in the identification and isolation of brain tumor stem cells. The correlation between CD133 expression in tumor tissues with patients survival is still controversial. CD133 expression is determinated by methylation status of the promoter region 1–3. Aberrant methylation of CD133 was observed in glioblastoma. To date, a direct link between CD133 methylation and patient outcome has not been established.To address this question, we studied CD133 expression and promoter methylation in a series of 170 gliomas of various grade and histology, and investigated the correlation of CD133 expression and promoter methylation with patient outcome.We detected five CD133 promoter methylation patterns in 170 glioma samples: methylation only (M+, U−), unmethylation only (M−, U+), both methylation and unmethylation equally (M+, U+), high methylation and low unmethylation (M+, Ul), and low methylation and high unmethylation (Ml, U+). By multivariate survival analysis, we found CD133 promoter methylation status was significant (P < 0.01) prognostic factors for adverse progression-free survival and overall survival independent of tumor grade, extent of resection, or patient age. CD133 immunostaining showed considerable variability among tumors. While, there was lack of correlation between CD133 protein expression and patient’s survival. Furthermore, no correlation between CD133 protein expression and CD133 promoter methylation status was observed (Kw = −0.165).CD133 promoter methylation status in glioma is closely correlated with patient survival, which suggest CD133 promoter methylaiton pattern is a promising tool for diagnostic purposes.

## Introduction

Increasing evidence suggests that there is a small subset of cells, called cancer stem cells (CSC), that are responsible for cancer initiation and development [1]. The CSC model of tumor development suggests that the clinical behavior of a tumor will be largely determined by a subpopulation of cells that are characterized by their ability to initiate new tumors [2]. Recently, CSC has been described in several solid tumors,including brain tumors [3, 4]. These studies used putative stem cell markers or side populations to isolate unique subsets of cancer cells from different types of tumors. These markers included CD24, CD44, CD133, and CD166 that are also expressed in normal cells. Among these markers, CD133 were widely used for isolating CSC from solid tumors [5–8].

Surface membrane protein CD133, which is normally expressed in a subset of putative neural stem/precursor cells in the normal brain, have been identified in brain tumors [4, 5, 9, 10]. CD133-positive tumor cells can initiate neurospheres, which exhibit self-renewal, differentiation, and proliferation resembling that of normal NSCs. The transplantation of CD133-positive tumor cells into nonobese diabetic/severe combined immunodeficient mice is sufficient to produce tumors phenotypically identical to the patient’s original tumors [4, 11]. CD133-positive glioma cells were also reported to possess enhanced chemoresistance and radioresistance, resulting in tumor progression and recurrence [12–15]. Therefore, the regulatory mechanisms of CD133 expression could help to elucidate the development of gliomas.

Changes in DNA methylation patterns are an important hallmark of tumor development and progression [16]. Although the role of hypermethylation in the silencing of tumor suppressor genes is now well-documented, abnormal methylation contributes to neoplastic progression in numerous types of human cancer, including glioblastomas [17–19]. CD133 expression in glioblastomas is determinated by methylation status of the promoter region 1–3 in vitro [20]. Strong methylation of CD133 promoter displayed in CD133-negative tumor cells, but less in the CD133-positive fraction [21]. Surprisingly, however, apart from the observation of aberrant methylation of CD133 in glioblastoma [21], a direct link between CD133 methylation and patient outcome thus far has not been established. To address this question, we studied CD133 expression and promoter methylation in gliomas, and investigated the correlation of CD133 expression and promoter methylation with patient outcome.

## Materials and methods

### Study sample

One hundred and seventy glioma samples used for this study were derived from formalin-fixed and paraffin-embedded surgical tissue samples obtained from the archive of Department of Neuropathology, Huashan Hospital, Fudan University, with approval from the institutional review board. This study compiles data for glioma of tumor stages II to IV (n = 170) and normal brain controls (n = 3). Three noncancer brain tissues used in this study were also obtained from the Neural Stem Cell Laboratory at Huashan Hospital, with institutional review board approval. Tumors were histopathologically classified according to the WHO classification. Informed consent was obtained from each patient according to the research proposals approved by the Institutional Review Board at the Medical Faculty Shanghai. Eligibility criteria included written informed consent and availability of tumor tissue and of follow-up data. Clinical information was obtained by reviewing the medical records on radiographic images, by telephone, and by review of death certificate. Patient data were analyzed after a mean follow-up period of 134.3 (±90.5) weeks. The demographic and clinical characteristics of the patients are showed in Table 1.

**Table 1:** Clinical characteristics of study sample

WHO grade	n	Sex (M/F)	Histology/WHO grade	n	Median age (y)	Median PFS (wk)	No tumor regrowth	Median OS (wk)	Alive at LO
II	62	40/22	A/2	55	41(2–70)	195^a^ (11–308)	41/55	196(2–308)	45/55
			O/2	7	48(9–65)	232 (84–310)	1/7	232(150–310)	1/7
III	29	18/11	AA/3	28	48.5(19–69)	69^b^ (6–306)	8/28	101(2–355)	8/28
			OA/3	1	22	145	0/1	175	1/1
IV	79	52/27	pGBM	73	53(11–79)	29^c^ (11–242)	6/73	66(2–242)	9/73
			sGBM	6	45.5(38–61)	14^d^ (9–24)	1/6	41(15–87)	0/6

*n* case number; *M* male, *F* female, *LO* last observation, *A* astrocytoma, *O* oligodendroglioma, *AA* anaplastic astrocytoma, *AO* anaplastic oligodendroglioma, *pGBM* primary glioblastoma, *sGBM* secondary glioblastoma

^a^PFS of two patients could not be assessed

^b^PFS of two patients could not be assessed

^c^PFS of thirteen patients could not be assessed

^d^PFS of one patients could not be assessed

### Methylation-specific PCR

Genomic DNA was isolated from paraffin embedded samples DNA was extracted using QIAamp DNA FFPE Tissue Kit(Qiagen). Bisulfite modification of genomic DNA was carried out using the EZ DNA methylation kit (Zymo Research). For PCR amplification, primer sequences for methylation-specific PCR (MSP) analysis were designed using MSP Primer. We performed methylation analysis of the CD133 promoter using MSP primer pairs covering the putative transcriptional start site in the 5 CpG island with 1 μL of bisulfite-treated DNA as template and ZymoTaq DNA polymerase (Zymo Research) for amplification, as previously described. The annealing temperature was 58 °C. All reactions were done twice to exclude unspecific PCR amplifications. Normal human lymphocyte DNA was used as negative control for methylated alleles of CD133, and placental DNA treated in vitro with SssI methyltransferase (New England Biolabs) was used as positive control. Controls without DNA were used for each set of MSP assay. PCR products were separated on 3 % agrose gels, stained with ethidium bromide, and examined under UV illumination. CD133 promoter methylation patterns were methylation only (M+, U−), unmethylation only (M−, U+), both methylation and unmethylation equally (M+, U+), high methylation and low unmethylation (M+, Ul), and low methylation and high unmethylation (Ml, U+). Investigators doing these assays were blinded to clinical information.

### Immunohistochemistry

Paraffin Sects. (5–7 μm) were stained for CD133 using a mouse monoclonal anti-CD133 antibody originally used to enrich tumorigenic CD133-positive cells from gliomas as well as an isotype IgG2b control antibody (both Miltenyi Biotec). Fixation and staining were carried out as described [22]. CD133 staining data were obtained from at least two sections per tissue. Immunohistochemically stained slides were reviewed by two investigators independent from one another and blinded to all clinical data. CD133 staining of the whole tissue section was semiquantitatively graded for percentage of cells stained in n.d. (not detectable) or <1, 1 to 10, 10 to 50, and >50 % CD133-positive cells per section.

### Correlation of CD133 IHC results with MSP results

Weighted kappa (Kw) was used to evaluate agreement between CD133 promoter methylation status and immunohistochemically evaluated CD133 expression in tumor cells. For this purpose, we categorized the immunohistochemical CD133 values in ordinal as previous described: n.d. (not detectable) or <1, 1 to 10, 10 to 50, and >50 %. Kw values lie between zero (absence of agreement) and 1 (absolute agreement). Observed values of Kw were considered satisfactory if equal to or greater than 0.80.

### Statistical analysis

Progression-free survival (PFS) was calculated from the date of surgery until the date of documented tumor recurrence or further growth of residual tumor and defined as ‘‘tumor regrowth’’. Overall survival was defined from the day of surgery until death of the patient. For patients who had not experienced recurrence or death at the time of last follow-up, PFS and overall survival (OS) were censored at the date of last follow-up. In case of impossible patient contact, the last date of visit was taken as provisional end point to allow statistical analysis. The association between PFS or OS and CD133 expression or CD133 promoter methylation status was calculated using log-rank tests and presented as Kaplan–Meier plots. Furthermore, a multivariate analysis was done by using Cox proportional hazards regression to determine the prognostic effect of CD133 expression, CD133 methylation status and potential clinical variables (age, WHO grade, and extent of resection) on OS and PFS. Backward selection applying a stopping rule based on the Akaike information criterion was used to exclude redundant or unnecessary variables. For continuous variables, the cutoff level chosen was their median value. CD133 immunohistochemistry was reclassified as <1 and >1 % (including low, moderate, and high staining) for statistical purposes. Hazard ratios (HR) and their corresponding 95 % confidence intervals (95 % CI) were computed to provide quantitative information about the relevance of results of the statistical analysis. The relationship between tumor CD133 expression and MSP results were evaluated by the Cohen’s weighted kappa statistics. All statistical analyses were performed using SAS9.2 statistical software (SAS Institute Inc., N.C.). Statistical significance was set at the level of P < 0.05.

## Results

### CD133 promoter methylation status and patient prognosis

**Fig. 1:**
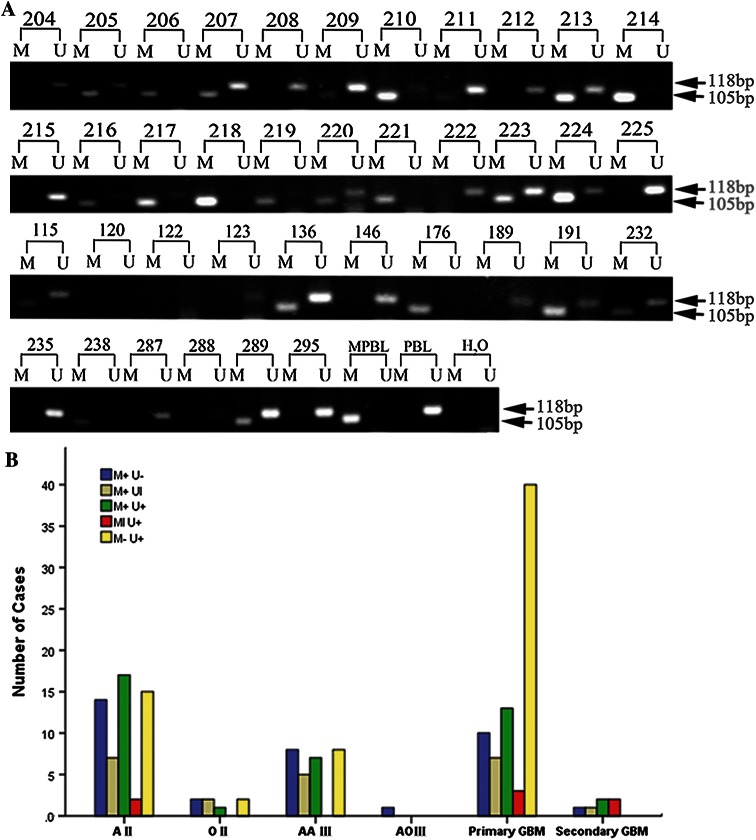
**a** Methylation analysis of the CD133 gene in primary glioma tissues. The MSP production of methylated and unmethylated CD133 are 105 and 118 bp, respectively. *M* methylation signal, *U* unmethylated signal, *MPBL* methylated peripheral blood lymphocyte DNA, *PBL* peripheral blood lymphocyte DNA, *ddH2O* water control adding no DNA. **b** CD133 methylation status in 170 gliomas differing in histology and WHO grade. CD133 promoter methylation patterns were methylation only (M+, U−), unmethylation only (M−, U+), both methylation and unmethylation equally (M+, U+), high methylation and low unmethylation (M+, Ul), and low methylation and high unmethylation (Ml, U+). *AII* astrocytoma grade II, *OII* oligodendroglioma grade II, *AAIII* anaplastic astrocytoma grade III, *AOIII* anaplastic oligodendroglioma grade III

We first investigated the CD133 promoter methylation status in three normal brain samples. As previous study showed [22], CD133 promoter were unmethylated in all normal brain using samples from noncancer patient tissue. Then, we explored the methylation pattern of CD133 promoter in different grade gliomas (Fig. 1). Methylation of the CD133 promoter was found in 106 of the 170 tumors (62.4 %). The distribution of CD133 methylation among the different patients’ characteristics is shown in Table 2. The presence of CD133 CpG island methylation in glioma patients was not associated with the sex (P = 0.26) or age of the patient (P = 0.19), but associated with the histological type of the tumor (P = 0.005) and grades (P = 0.005). In low-grade tumors (WHO grade 2), the percentage of methylated and unmethylated CD133 were 74.2 % (46 of 62) and 25.8 % (16 of 62), respectively. With the progression to GBM (WHO grade 4), the percentage of methylation decreased to 49.4 % (39 of 79), and the percentage of unmethylation increased to 50.6 % (40 of 79).

**Table 2:** The CD133 methylation pattern of the glioma patients

Variable	CD133 Methylaiton		*p*
	U	M	
Sex			
Male	38	72	
Female	26	34	0.26
Age			
< 45	23	51	
45–59	27	41	
≥ 60	14	14	0.19
Histology			
Astrocytoma	22	61	
Oligodendroglioma	2	6	
GBM	40	39	0.005
Grade			
II	16	46	
III	8	21	
IV	40	39	0.005

**Fig. 2:**
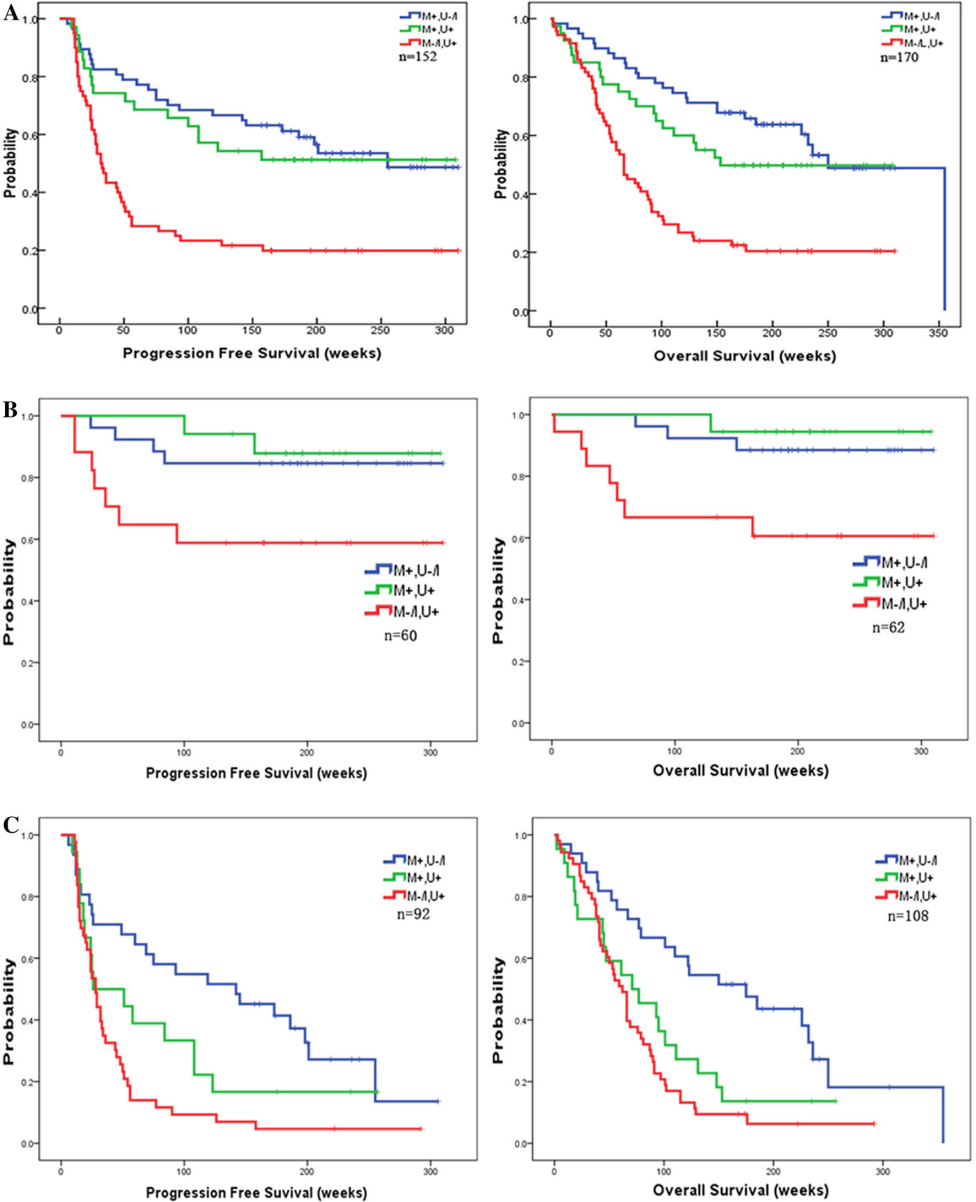
Kaplan–Meier plots showing a correlation of CD133 methylation status with PFS and OS. **a** The Kaplan–Meier plots of CD133 methylation status. The PFS and OS of patients with unmethylated CD133 promoter was 91.0 weeks (95 % CI, 61.3–120.8) and 113.7 weeks (95 % CI, 87.1–140.3), respectively. While, patients with methylated CD133 promoter showed a tendency to an increased PFS (189.7 weeks, 95 % CI, 164.1–215.3) and OS (218.9 weeks, 95 % CI, 200.4–271.6). **b** The Kaplan–Meier plots of CD133 methylation status in LGG. The PFS and OS of LGG patients with unmethylated CD133 promoter was 197.1 weeks (95 % CI, 132.5–261.8) and 209.6 weeks (95 % CI, 150.1–269.1), respectively. While, patients with methylated CD133 promoter showed a tendency to an increased PFS (271.0 weeks, 95 % CI, 235.7–306.3) and OS (286.2 weeks, 95 % CI, 260.5–311.9). **c** The Kaplan–Meier plots of CD133 methylation status in HGG.The PFS and OS of HGG patients with unmethylated CD133 promoter was 47.7 weeks (95 % CI, 29.3–66.1) and 77.1 weeks (95 % CI, 58.7–95.4), respectively. While, patients with methylated CD133 promoter showed a tendency to an increased PFS (139.5 weeks, 95 % CI, 100.5–178.5) and OS (172.2 weeks, 95 % CI, 128.8–215.7)

To investigate the effect of CD133 promoter methylation status on patient outcome, corresponding PFS and OS data were assessed from the study sample. PFS could not be assessed in eithteen. PFS and OS depending on various clinical variables and CD133 methylation status are summarized in Table 3. In univariate analyses, methylation of the promoter was positively correlated with PFS and OS. The PFS and OS of patients with unmethylated CD133 promoter was 91.0 weeks (95 % CI, 61.3–120.8) and 113.7 weeks (95 % CI, 87.1–140.3), respectively. While, patients with methylated CD133 promoter showed a tendency to an increased PFS (189.7 weeks, 95 % CI, 164.1–215.3) and OS (218.9 weeks, 95 % CI, 200.4–271.6). Such analysis indicated a strong correlation between CD133 promoter methylation status and both overall (P = 0.002) and progression-free (P < 0.001) survival (Fig. 2), suggesting that CD133 methylation of tumorigenic cells is associated with a more favorable prognosis. The importance of CD133 methylation as a prognostic factor was next determined by the Cox proportional hazards model analysis. Multivariate analysis confirmed CD133 methylation (HR 2.87; 95 % CI, 1.74–4.73; P < 0.001) as significant prognostic factors for longer OS, independent of WHO grade, age, and extent of resection; similar results were obtained for PFS and methylation of CD133 (HR 2.61; 95 % CI, 1.57–4.34; P < 0.001).

**Table 3:** Multivariate analysis of prognostic factors as covariables with CD133 methylaiton status or expression for glioma outcome

Variable	PFS		OS	
	HR (95 % CI)	p	HR (95 % CI)	p
CD133 methylation status	n = 152		n = 170	
Unmethylation	1		1	
Methylation	2.61 (1.57–4.34)	<0.001	2.87 (1.74–4.73)	<0.001
WHO grade				
WHO2	1		1	
WHO3	4.49 (2.14–9.38)	<0.001	7.06 (3.34–14.91)	<0.001
WHO4	7.74 (3.90–15.33)	<0.001	11.51 (5.86–22.61)	<0.001
Patient age	1.021(1.00–1.04)	0.016	–	0.054
Extent of resection	0.72 (0.55–0.94)	0.016	1.40 (0.71–2.76)	0.330

Backword stepwise (likelihood ratio) Cox regression model

Furthermore, we have investigated the effect of CD133 promoter methylation status on patient outcome by stratifying with tumor grades, corresponding PFS and OS data were assessed in LGG (low grade glioma, WHO 2) and HGG (high grade glioma, WHO 3 or 4) patients. PFS could not be assessed in two patients in LGG, and 16 patients in HGG. The PFS and OS of LGG patients with unmethylated CD133 promoter was 197.1 weeks (95 % CI, 132.5–261.8) and 209.6 weeks (95 % CI, 150.1–269.1), respectively. While, patients with methylated CD133 promoter showed a tendency to an increased PFS (271.0 weeks, 95 % CI, 235.7–306.3) and OS (286.2 weeks, 95 % CI, 260.5–311.9). Such analysis indicated a strong correlation between CD133 promoter methylation status and both overall (P = 0.008) and progression-free (P = 0.035) survival, suggesting that CD133 methylation of tumorigenic cells is associated with a more favorable prognosis in LGG patients. The PFS and OS of HGG patients with unmethylated CD133 promoter was 47.7 weeks (95 % CI, 29.3–66.1) and 77.1 weeks (95 % CI, 58.7–95.4), respectively. While, patients with methylated CD133 promoter showed a tendency to an increased PFS (139.5 weeks, 95 % CI, 100.5–178.5) and OS (172.2 weeks, 95 % CI, 128.8–215.7). Such analysis indicated a strong correlation between CD133 promoter methylation status and both overall (P < 0.01) and progression-free (P < 0.01) survival, suggesting that CD133 methylation of tumorigenic cells is also associated with a more favorable prognosis in HGG patients.

### Degree CD133 expression in glioma tissues

**Fig. 3:**
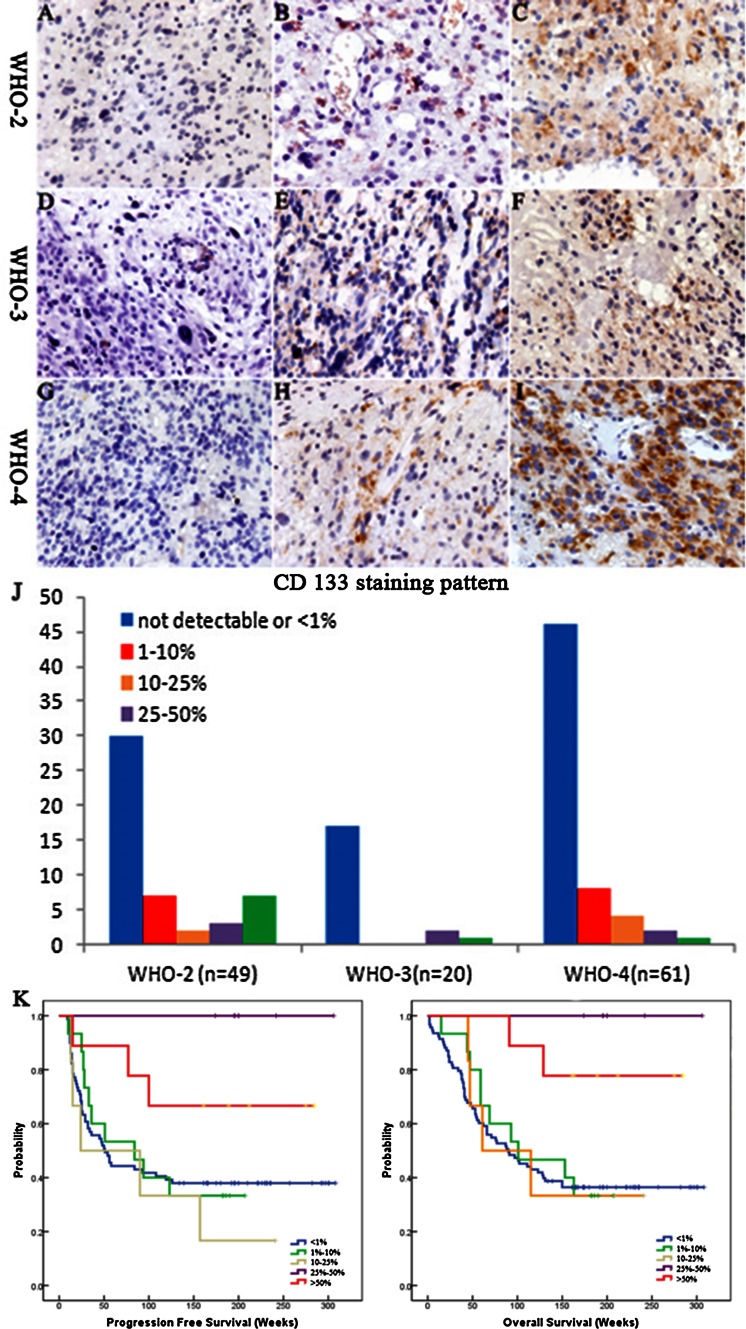
CD133 staining was also observed in dispersed single cells, a few large pleomorphic tumour cells and a few mitotic cells(**a**–**i**). CD133 immunostaining showed considerable variability among tumors ranging from complete lack of immunoreactivity (**a**, **d**, **g**) to expression in single cells (**b**, **e**, **h**) or staining of cell clusters (**c**, **f**, **i**). Scale bar 25 μm. **j** CD133 staining pattern in 130 gliomas differing in histology and WHO grade. **k**, **l** Kaplan–Meier plots showing no correlation of CD133 staining pattern with PFS (**k**) and OS (**l**)

Expression of the CD133 antigen was assessed by immunohistochemistry in paraffin-embedded sections in a panel of 130 gliomas of different WHO grades and histologies. CD133 immunostaining showed considerable variability among tumors ranging from complete lack of immunoreactivity (Fig. 3a, d, g) to expression in single cells (Fig. 3b, e, h) or staining of cell clusters (Fig. 3c, f, i). CD133 positive immunostaining was detected in 37 tumors (28.5 %). In all positive tumor samples, heterogenous immunostaining was observed; areas with complete loss of CD133 expression alternated with areas of scattered or clustered cells with strong immunoreactivity. Surprisingly, further analysis showed there were no correlations between CD133 expressions with WHO grades (Fig. 2). CD133 negative and <1 % of tumor cells were predominant in all grades of gliomas. 24.5 % (12/49) of grade 2 gliomas were found to express CD133 over 10 %. With progression to anaplastic gliomas (WHO grade 3) and GBM (WHO grade 4), the percentage of CD133 over 10 % decrease to 15.0 % (3 of 20) and 11.5 % (7 of 61), which seems opposite to grade of gliomas.

To investigate the effect of proportion of CD133 positive cells on patient outcome, corresponding PFS and OS data were assessed from the study sample. Univariate analysis documents there was no correlation of PFS and OS with the numbers of CD133-positive cells (Fig. 3k, l).

### CD133 promoter methylation status and cd133 protein expression

**Fig. 4:**
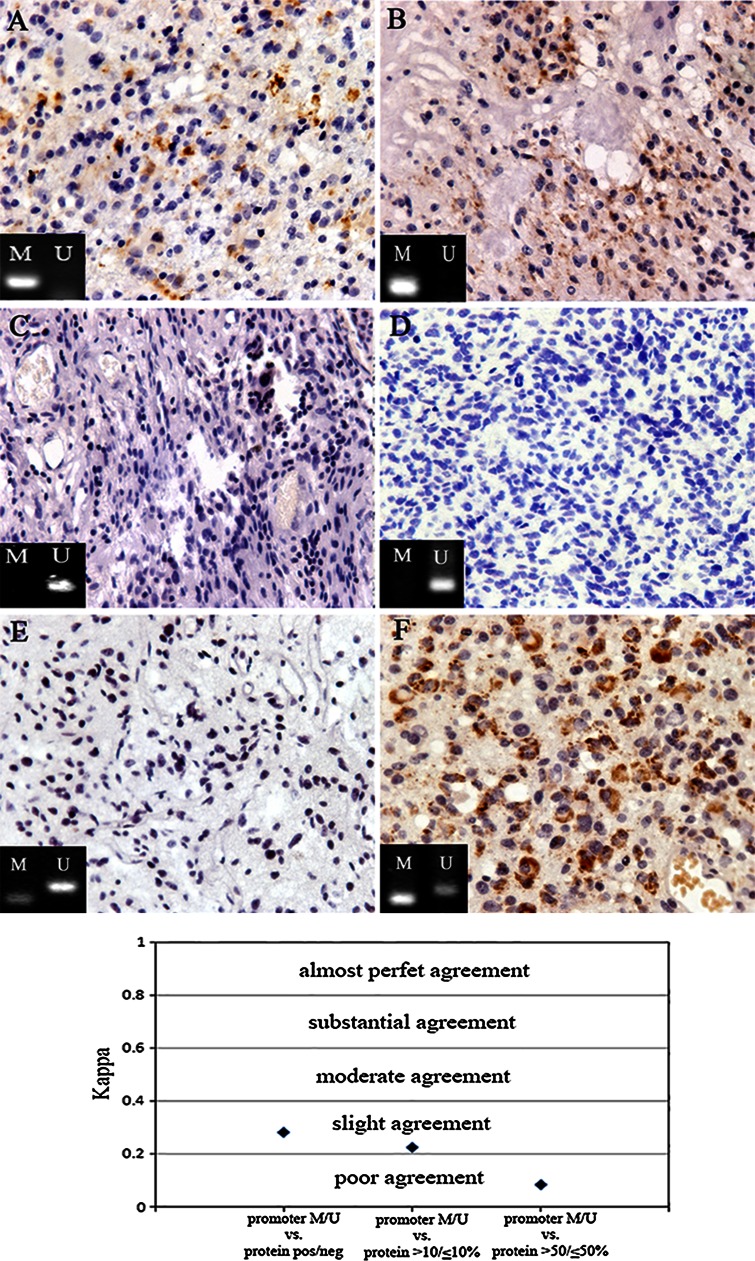
No correlation between CD133 protein expression and CD133 promoter methylation status. **a**, **b** CD133 promoter was totally methylated, some degree of CD133 immunoreactivity was still detected. **c**, **d** CD133 expression was completely lost, CD133 promoter was universally unmethylated. **e**, **f** CD133 expression was either completely negative or over 50 % in those samples of which CD133 promoter methylation pattern was both methylation and unmethylation. The relationship between tumor CD133 expression and MSP results were evaluated by the Cohen’s weighted kappa statistics, which showed no correlation between CD133 protein expression and CD133 promoter methylation status (Kw = −0.165)

After demonstrating there was lacking of correlation between CD133 protein expression and histological grades, we investigated relationship of CD133 protein expression and CD133 MSP(methylated or unmethylated) in glioma samples(Fig. 4). We categorized the immunohistochemical CD133 values in three different ways (see also Materials and Methods): (i) CD133 negative (<1 %) versus CD133 positive; (ii) Low (>1 and <10 %) versus high (>10 and <50 %) CD133 expression; (iii) CD133 expression in <50 % of tumor cells versus CD133 expression in >50 % of tumor cells. We found poor to slight agreement between MSP and CD133 IHC at all categories (Kw = −0.165).

## Discussion

CD133 has played a pivotal role in the identification and isolation of brain tumor stem cells. However, the correlation between CD133 expression in tumor tissues with patients survivals is still controversial [23, 24]. This study represents, to our knowledge, the first investigation aimed at evaluating the prognostic significance of CD133 in a series of patients with gliomas in which both tumor CD133 expression and promoter methylation status were simultaneously evaluated. Our results show that CD133 promoter methylation is independently associated with a longer overall survival in patients with anaplastic gliomas, whereas CD133 expression in IHC has no prognostic implications. The strength of these results relies mainly on the fact that CD133 predictive value has been evaluated after adjusting this variable for well-recognized clinicopathologic prognostic factors.

In our study, CD133 methylation pattern was heterogeneous, ranging from unmethylation to methylation. Differences in the methylation status of the CD133 promoter in tumor cell subpopulations may explain this heterogeneity. Other potential explanations for this variability include monoallelic promoter methylation, or loss of heterozygosity in 4p15. Whereas the presence of contaminating normal cells may not be ruled out. The presence of CD133 CpG island methylation in glioma patients was associated with grades of glioma. In low-grade tumors (WHO grade 2), the percentage of methylated and unmethylated CD133 were 74.2 % (46 of 62) and 25.8 % (16 of 62), respectively. With the progression to high grade glioma (WHO grade 3 and 4), the percentage of methylation decreased to 44.4 % (62 of 108), and the percentage of unmethylation increased to 55.6 % (60 of 108). This findings was consistent with the others study, which reported that promoter methylation of CD133 was lower in advanced colorectal carcinomas and aggressive breast cancer [25, 26]. This is concordant with the biologically aggressive nature of high grade glioma, poor overall prognosis, and limited therapeutic targets. More important, our study first reported that the methylation status of the CD133 promoter may have prognostic value. We found CD133 hypermethylation to be significantly (almost two-fold) more common in long-term survivors (74 %) as compared to in short-term survivors (43 %) not only in high grade glioma, but in low grade glioma. Multivariate analysis indicated CD133 methylation was a significant prognostic factor in gliomas, independent of tumor grade, extent of resection, and patient age. This findings may suggest that methylation of CD133 promoter may directly relevant to the stem cell state of CSC. In recent study, Gopisetty et al. demonstrate a role for the promoter CpG island in critically regulating CD133 expression in glioma stem cell (GSC) [27]. When the GSC cells were sorted into CD133+ve and −ve fractions, CD133+ve cells are significantly resistant to chemotherapeutic agents compared with autologous CD133−ve cells and to radiation therapy. CD133 promoter was hypermethylated in CD133−ve GSC and glioma cells, but unmethylated in CD133+ve ones. Promoter methylation of CD133−ve fraction can be reversible by demethylation agents and reproduced CD133 strongly supporting a functional role for methylation in the repression of CD133.

In the present study, a heterogeneous staining pattern of CD133 was observed within the individual tumor and between different tumors of the same grade. This is in agreement with other studies which show expression of CD133 was considerable various in gliomas on frozen section [23, 28] and by flow cytometry [4]. We found that 85.0 % of the anaplastic astrocytomas and 75.4 % of the glioblastomas were negative for CD133, which was much lower than that of other studies who found that less than 40 % of anaplastic gliomas and less than 2 % of glioblastomas were negative for CD133 [23, 24]. Use of different CD133 antibody clones might explain the difference between our findings and the results obtained in other studies. In other studies, the monoclonal antibody clone AC133 and W6B3C1 were from Miltenyi Biotec [3, 29], and goat polyclonal antibody was from Santa Cruz were used [28], whereas we used the monoclonal antibody clone CD133-1 from Miltenyi Biotec. Another explanation was use of different tissue fixation methods. In other studies, cryosections were used to stain CD133, whereas we used formalin-fixed paraffin sections because the morphology is better preserved in paraffin sections making it easier to evaluate a membrane staining like CD133 [29].

Comparing the CD133 expression assessed by ICH with overall patient, we found CD133 protein expression in glioma was insufficient correlated with patient survival. Therefore, in our study, CD133 IHC does not prove to be a clinically usable tool in the prognostic assessment of glioma. Previously published studies on diffuse glioma [23, 30, 31] reported a significant association of immunohistochemically assessed CD133 expression and patient survival. However, this association was not confirmed our present study in a larger patient cohort. This result may be explained by the bona fide cancer stem cell being a subpopulation of the CD133+ tumor cells, but it is also likely that other non tumor stem cells apart from endothelial cells express CD133. Other explanations may be methodological differences (e.g., different pretreatment of sections prior to immunostaining and different type of sections (paraffin sections vs. frozen sections) [23].

Very few studies have investigated the relationship between CD133 promoter hypermethylation and protein expression in human glioma samples. In the present study, there was an inconsistent correlation between aberrant promoter methylation and loss of protein expression. To date, this inconsistency was not limited to the CD133 gene [32–35]. Promoter methylation is clearly involved in the inactivation of CD133 gene in numerous tumors and cancer cell lines [20, 21], but regulation of CD133 expression is a more complex phenomenon in which abnormal methylation of the promoter is not the only determining factor [21, 36, 37]. Indeed, several studies indicate that grade of methylation both in the promoter region and in neighboring sequences may regulate gene expression [20, 37, 38]. As discussed previously, another possible explanation for this finding is CD133 protein expression in entrapped pre-existing endothelial cells, which is confirmed by flow cytometry showing that CD133+ cells could have either blood vessel or glioma origin [39, 40]. Therefore, there is increasing evidence that immunohistochemically assessed CD133 expression is a poor indicator of CD133 promoter methylation status in glioma.

However, the importance of this study goes beyond showing the putative clinical benefit of CD133 methylation status as a prognositic marker. Currently, little data exist on the clinical relevance of CSCs. Properties of CSCs could well explain many clinical features of cancer, such as recurrence, and therapy resistance [1, 41–49]. Some studies also tested whether the prevalence of putative CSCs in the tumor was relevant to patient outcome, but the results were controversial [23, 24, 30, 31, 50–54]. In light of the results reported here, the discrepancy may seem not surprising because different CSC markers, heterogenous expression, and observation variability all effect on the results. Here, for the first time, we presented a direct link between the methylation of a CSC marker and patients’ outcome. These data provide strong supportive evidence for the CSC model and the clinical relevance of the CD133 methylation status in gliomas.

To conclude, in our study, CD133 promoter methylation status in glioma is closely correlated with patient survival, which suggest CD133 promoter methylation pattern is a promising tool for prognostic purposes. While, lack of association with CD133 IHC and with patient survival impede the use of CD133 IHC as a clinically useful biomarker for routine purposes and clinical decision making.
